# Antibiotic, Heavy Metal, and Biocide Concentrations in a Wastewater Treatment Plant and Its Receiving Water Body Exceed PNEC Limits: Potential for Antimicrobial Resistance Selective Pressure

**DOI:** 10.3390/antibiotics12071166

**Published:** 2023-07-09

**Authors:** Kelechi B. Chukwu, Ovokeroye A. Abafe, Daniel G. Amoako, Sabiha Y. Essack, Akebe L. K. Abia

**Affiliations:** 1 Antimicrobial Research Unit, College of Health Sciences, University of KwaZulu-Natal, Durban 4000, South Africa; abafeo@ukzn.ac.za (O.A.A.); amoakod@ukzn.ac.za (D.G.A.); essacks@ukzn.ac.za (S.Y.E.); 2 Residue Laboratory, Agricultural Research Council—Onderstepoort Veterinary Research Campus, Onderstepoort 0110, South Africa; 3 School of Geography, Earth and Environmental Sciences, University of Birmingham, Birmingham B15 2TT, UK; 4 Department of Integrative Biology and Bioinformatics, University of Guelph, Guelph, ON N1G 2W1, Canada; 5 Environmental Research Foundation, Westville 3630, South Africa

**Keywords:** environmental stressors, antimicrobial resistance, selective pressure, biocides, heavy metals, antibiotic residues, aquatic environment, PNEC

## Abstract

Although the rise in antimicrobial resistance has been attributed mainly to the extensive and indiscriminate use of antimicrobials such as antibiotics and biocides in humans, animals and on plants, studies investigating the impact of this use on water environments in Africa are minimal. This study quantified selected antibiotics, heavy metals, and biocides in an urban wastewater treatment plant (WWTP) and its receiving water body in Kwazulu-Natal, South Africa, in the context of the predicted no-effect concentrations (PNEC) for the selection of antimicrobial resistance (AMR). Water samples were collected from the WWTP effluent discharge point and upstream and downstream from this point. Heavy metals were identified and quantified using the United States Environmental Protection Agency (US EPA) method 200.7. Biocides and antibiotic residues were determined using validated ultra-high-performance liquid chromatography with tandem mass spectrometry-based methods. The overall highest mean antibiotic, metal and biocide concentrations were observed for sulfamethoxazole (286.180 µg/L), neodymium (Nd; 27.734 mg/L), and benzalkonium chloride (BAC 12) (7.805 µg/L), respectively. In decreasing order per sampling site, the pollutant concentrations were effluent > downstream > upstream. This implies that the WWTP significantly contributed to the observed pollution in the receiving water. Furthermore, most of the pollutants measured recorded values exceeding the recommended predicted no-effect concentration (PNEC) values, suggesting that the microbes in such water environments were at risk of developing resistance due to the selection pressure exerted by these antimicrobials. Further studies are required to establish such a relationship.

## 1. Introduction

The current high use of antimicrobials in humans, animals and plants has spilt over into the environment in the form of antimicrobial residues with the subsequent selection pressure for the emergence and proliferation of drug-resistant microorganisms and antimicrobial resistance genes (ARGs) [1,2]. Furthermore, emissions from industrial-scale production of antimicrobials expose environmental bacterial communities to unprecedented selection pressures where concentrations exceed the predicted no-effect concentrations PNEC), resulting in the rapid development of antimicrobial resistance (AMR) [3].

Antimicrobials such as antibiotics, heavy metals and biocides are introduced into the environment via discharges from pharmaceutical industries, hospital effluents, direct excretion from humans and livestock, and runoff from farms [4]. Antibiotics that have been reported in wastewater and surface water bodies include sulfamethoxazole (SMX), tetracycline (TET), erythromycin (ERY), ciprofloxacin (CIP), amoxicillin (AMX), and trimethoprim (TMP) [4,5,6,7,8,9]. For example, in hospital wastewater in Ghana, the most detected antibiotics were CIP (15 µg/L) and SMX (7.2 µg/L) [10], while in Tunisia, over 1100 µg/L of erythromycin was detected [11].

Heavy metals in the environment could originate from geological sources like weathering of rocks and soil deposition of particulates. However, elevated concentrations usually come from anthropogenic sources such as industry, mining, and domestic discharges. Most of these heavy metals end up in surface waters (rivers and estuaries) or sediments, creating environmental reservoirs of these pollutants [12]. Many studies have reported varying concentrations of heavy metals in aquatic systems in different parts of Africa. For example, copper was detected at concentrations reaching 1070 µg/L in river water in Ethiopia [13]. Similarly, Diop et al. (2015) investigated the presence of heavy metals in coastal and estuary sediments in Dakar, Senegal, and recorded up to 1308 mg/kg of lead in sediments [14]. Furthermore, in South Africa, a study found aluminium concentrations ranging between 1.01–9.644 mg/L (water) and 4296–5557 mg/kg (sediments) in the Mvudi River [15], while another study in a wastewater treatment plant recorded high values of iron (69.789 mg/kg in sludge) and copper (6.588 mg/L in wastewater), exceeding local and international standards [16].

The Biocide Product Directive 98/8/EC of the European Commission describes biocides as “active substances or preparations containing one or more active substances used directly or in forms that can be applied or supplied to destroy, render harmless, prevent the action or otherwise exert a controlling effect on harmful organisms, by either chemical or biological means” [17,18,19]. While some biocides like hexachlorophene that causes skin disorder have been prohibited, others like triclosan are still widely used in diverse consumer and healthcare products, such as soaps, scrubs, gels, toothpaste, deodorants, hospital, and disinfectants. Similarly, benzalkonium chloride (BAC), a quaternary ammonium compound (QAC), is used extensively as an active ingredient in preservatives, medical disinfectants and ophthalmic systems [20,21,22,23,24,25]. These substances have also been reported in the environment. Studies have detected various biocides in varying concentrations in different parts of the world, ranging from 50 ng/L in Germany to 13 µg/L in South Africa [23,26,27,28,29,30,31,32].

Concentration limits have been set for these substances in different environments by governments and regulatory agencies, and their concentrations are expected to be below these acceptable limits. These limits are mostly set as predicted no-effect concentration (PNEC), which are levels presumed to exert no selection pressure on bacteria from the substances [1,33]. However, these substances are frequently discharged into the environment at concentrations greater than these limits, especially in most low- and middle-income countries, where treatment facilities function sub-optimally or are completely absent. The harmful effects and the potential to trigger antimicrobial resistance warrant the constant monitoring of these pollutants, especially in aquatic environments. Furthermore, studies addressing this topic are limited in Africa. With compromised sanitation facilities, these chemicals could easily find their way into different environments, including water bodies used by communities in resource-poor settings without access to potable water. Therefore, this study evaluated the presence and concentrations of selected antibiotics, heavy metals, and biocides in a WWTP’s effluent and its receiving water body in KwaZulu-Natal, South Africa. KwaZulu-Natal is the second largest province in South Africa, with a population of close to nine million inhabitants, living in a mix of rural, periurban and highly industrialised areas. Thus, the province represents the typical demographics of the country. In addition, the observed concentrations were compared to existing limits to infer their potential to exert selection pressure on environmental bacteria, leading to AMR.

## 2. Results

### 2.1. Distribution of Heavy Metals

**Figure 1:**
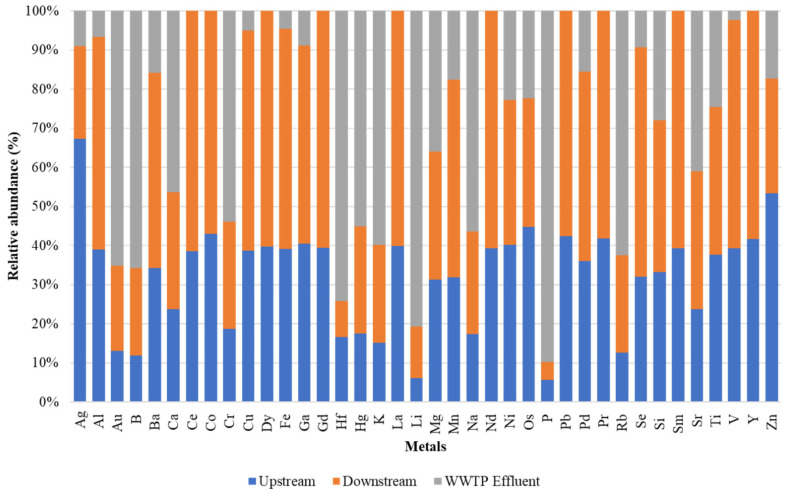
Heavy metal distribution across sampling points.

Thirty-eight metals were detected across all the samples (Figure 1). Of these, the highest mean metal concentration was observed for Nd (27.734 mg/L), while the least was for Fe (0.001 mg/L) (Table S1; Supplementary Material). Summing up the metal concentrations at each of the three sampling points over the entire study period, there was an overall higher combined metal concentration in the effluent samples (178,473 mg/L) than in the downstream (105,354 mg/L) and upstream (35,071 mg/L) samples. However, only Ca (*p* = 0.038), Mg (*p* = 0.002), Ni (*p* = 0.025), Os (*p* = 0.036), Si (*p* = 0.009), Sr (*p* = 0.022), and Ti (*p* = 0.017) were statistically significantly different across the sites (Table S2; Supplementary Materials).

### 2.2. Distribution of Antibiotics

A total of 13 antibiotics were detected across the samples, with sulfamethoxazole being the most detected (286.180 µg/L) and penicillin being the lowest (2.2 µg/L) (Table 1; Table S3; Supplementary Materials).

Overall, the highest total antibiotics concentration was observed in the WWTP effluent samples (1335.400 µg/L), followed by downstream (846.830 µg/L) and upstream (724.120 µg/L) (Table S3, Supplementary Materials), with the difference being statistically significant for all the antibiotics identified except for Sulfamonomethoxine (*p* = 0.131) and penicillin (*p* = 0.131) (Table S4; Supplementary Materials). Some antibiotics, like tetracycline and sulfapyridine, were only detected in effluent and downstream samples.

### 2.3. Distribution of Biocides

Benzalkonium chloride (BAC) 12 had the highest mean concentration (7.805 µg/L), while the least detected was BenthEZ (0.035 µg/L) (Table S5; Supplementary Materials). However, the single highest concentration (29.58 µg/L) was recorded in the upstream sample in the afternoon sampling period. Additionally, BAC14 was only detected in the downstream samples, while BAC14 and DDAC were not detected in the upstream samples.

## 3. Discussion

This study quantified selected antibiotics, heavy metals, and biocides in an urban WWTP and its receiving water body in Kwazulu-Natal, South Africa, comparing them to the PNEC for the selection of AMR. Heavy metals were identified and quantified using the US EPA method 200.7. Biocides and antibiotic residues were determined using validated ultra-high-performance liquid chromatography with tandem mass spectrometry-based methods. The highest antibiotic, metal and biocide concentrations were observed for sulfamethoxazole, neodymium, and BAC 12, respectively. In decreasing order per sampling site, the pollutant concentrations were effluent > downstream > upstream. Most of the pollutants measured recorded values exceeding the recommended PNEC values.

### 3.1. Heavy Metals

A total of 69 heavy metals were screened, of which 26 heavy metals were detected, with seven having concentrations above the recommended World Health Organization (WHO) (WHO 2008) and South African National Standard (SANS) [34] limits. Although Al (3.43 mg/L) and Zn (0.078 mg/L) were higher than the SANS limits (Al—0.3 mg/L; Zn—0.005 mg/L), other heavy metals (As, Ag, Au, Ba, Be, Cd, Co, Cr, Hg, Mo, Ni, Pb, Sb, Se, U, V) were detected at very low concentrations (Table S2). Previous South African studies had reported low heavy metal concentrations in some South African aquatic ecosystems and suggested that the studied water bodies were not heavily impacted, especially by industries and metal works [15,35]. However, for some heavy metals, like Cd, Pb, Al, Fe, and As, the concentrations reported were lower than those reported in previous studies [36,37]. The differences could be due to the anthropogenic activities surrounding the various water bodies in the different studies. High Cd and As environmental concentrations, for example, have been associated with the heavy use of agricultural chemicals like in fish farming [38], which were not practised in our study area. Additionally, this study only focused on surface water, while Letsoalo et al. [37], for example, analysed both surface and sediment samples (sediments can absorb more heavy metals), which could have increased their chances of isolating higher metal concentrations.

Comparing the different sampling sites in this study revealed that the WWTP effluent samples had a statistically significantly higher heavy metal concentration than the downstream and upstream samples. This could be associated with reduced treatment plant efficiency in removing inorganic pollutants. Similar findings had previously been reported in South Africa. For example, Agoro et al. [16], in a study conducted on the evaluation of heavy metals in wastewater and sludge in selected WWTPs in the Eastern Cape Province, South Africa, reported that the removal efficiency for heavy metals like Cu and Zn was very low in all studied WWTPs, resulting in poor wastewater effluent quality regarding heavy metals. The authors concluded that this resulted in contamination of the downstream sites and suggested that the effluent was unsuitable for irrigation. The downstream site in this study also had a higher metal concentration than the upstream site, indicating that the WWTP significantly influenced the presence of pollutants in the receiving water body. Although in our study, we observed low concentrations of most heavy metals downstream compared to the SANS values, several authors have indicated the ability of sub-inhibitory concentrations of heavy metals to select for resistance genes in microbes and that they could still be toxic to humans and other aquatic organisms [16,37,39]

Heavy metals, even at low concentrations, can alter bacterial efflux pump system expression, promoting cross-resistance to antibiotics and contributing to the development of multidrug-resistant bacterial species [39]. For example, in a laboratory experiment to determine the effect of sub-lethal concentrations of two heavy metals (Cu and Zn) on the development of multiple antibiotic resistance in bacteria in liquid media, Xu et al. [39] observed that exposure of *E. coli* to these heavy metals resulted in the upregulation of efflux pump genes that also favoured antimicrobial resistance. Similarly, an earlier study on river water exposed to nickel or cadmium stressors resulted in increased antibiotic-resistant bacteria [40]. Li et al. [41] observed that sub-minimum inhibitory concentrations of silver (Ag^2+^), zinc (Zn^2+^) and copper (Cu^2+^) increased the mutation rates of exposed microbes, and the mutants exhibited significant resistance to multiple antibiotics. Furthermore, Chen et al. [42] observed that the *sul*2 gene responsible for resistance to sulphonamides co-occurred with metal resistance genes in their studied bacteria, suggesting a co-selection of resistance. Based on the above studies, it could be speculated that the sub-lethal heavy metal concentrations recorded in this study might present a potential for antimicrobial resistance development in the studied environment. However, such speculations could be verified through exposure experiments to the observed environmental concentrations to ascertain if such exposure would induce phenotypic and genotypic AMR in environmental bacteria within the studied aquatic ecosystem.

### 3.2. Antibiotics

**Figure 2:**
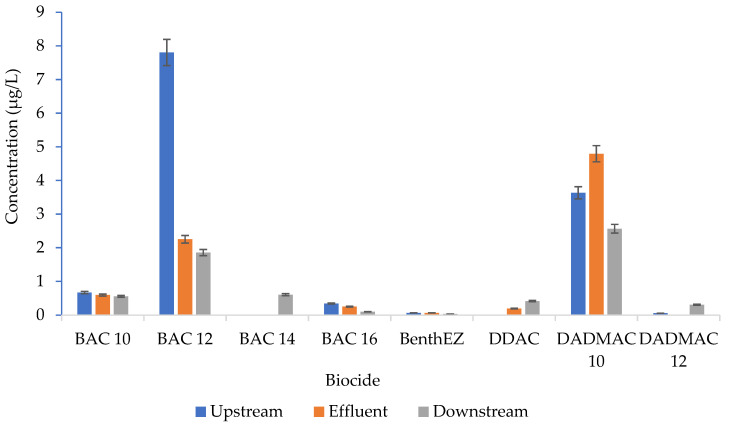
The overall distribution of biocide across sampling sites. Error bars represent the standard error of the mean.

Antibiotics are extensively used in human and animal medicine for treatment and prophylaxis. However, approximately 75 to 95% of the antibiotics used in food animals are excreted unmetabolised or partially metabolised [43]. As a result, these antibiotics and their residues end up in waterways, mainly through WWTPs, and it has been shown that WWTPs are hotspots for the dissemination of antibiotics into the environment, especially aquatic ecosystems [44,45,46]. Due to their potential to adversely affect human, animal and environmental health, international (PNEC) and local (SANS) guideline values have been set for allowable environmental concentrations. In this study, a total of 24 antibiotics were targeted, and we detected 14 across all samples, with the WWTP effluent samples recording the overall highest total antibiotic concentration compared to the other sites. This confirms the role of WWTPs as hotspots for the discharge of antibiotics into receiving water, as previously mentioned. This argument is further strengthened by the higher antibiotic concentrations observed in the downstream samples compared to the upstream samples (Figure 2; Table S3).

Sulfapyridine is a sulphonamide with allergenic properties and a by-product of sulfasalazine. The use and manufacture of this drug ended in 1990, and it is a known environmental pollutant [47]. However, the drug is still used in veterinary medicine to treat diarrhoea in dogs [48]. Sulfapyridine is not registered for use in South Africa (www.sahpra.org.za), indicating that its identification in this study, like in another recent South African study, suggests possible illegal use within the country [49]. Sulfamethoxazole is widely used in treating chronic bronchitis, urinary tract infections, enteric infections as well as pneumocystis pneumonia (PCP), which is an opportunistic infection in immunocompromised people such as HIV patients; it is also used in livestock animals treatment [50].

In reviews conducted on antibiotics in African waters [7,50,51], it was reported that sulfamethoxazole was the most detected drug, as was the case in this study. This can be explained by the fact that South Africa has a high HIV prevalence rate, estimated at over 13%, translating to over 8 million people living with the infection [52], thus the extensive use of the drug within the country for PCP prophylaxis.

Most of the antibiotic concentrations detected in our study were comparable to those detected across Africa [9,50,53,54,55,56]. However, other antibiotics like tetracycline, doxycycline, amoxicillin, and lincomycin were detected at concentrations higher than those reported in other African countries [7,57,58]. Most importantly, some of these concentrations were higher than the recommended PNEC values, as observed for sulfamethoxazole tetracycline, oxytetracycline, lincomycin, and amoxicillin (Table 1). These high values indicate that the water environment may facilitate the selection of antimicrobial resistance in microbial communities. However, further studies would be required to establish such risk.

### 3.3. Biocides

Biocides have been widely used in different applications such as human medicine [59], water distribution pipelines to prevent microbially associated corrosion [60], water chemistry [61], antibiofilm coatings [62], and domestic/household products [63]. This has therefore led to their continuous discharge into the environment. Eight of the 10 targeted biocides detected in this study had quantifiable values (Table S5). Benzalkonium chloride (BAC) 12 had the highest detected concentration, while BenthEZ was the most detected in 75% of the samples. BAC 14 and DDAC were only detected in the effluent and downstream samples, again pointing to the role of WWTPs in surface water pollution. Additionally, the BAC 12 concentrations were higher than those reported in other studies [32,64,65,66,67,68]. This can be explained by the extensive use of BAC and its derivatives in detergents, disinfectants, hair shampoos and most personal care products [20,21,22,23,24,25]. Due to their potential deleterious effects, biocides have been included with detergents and regulated in South Africa by the National Regulator for Compulsory Specifications (NRCS) [69].

The concept of co-resistance is relatively broad depending on the specific study, although in its basic forms, it would imply the ability of an organism to harbour multiple resistance traits [70]. On the other hand, cross-resistance is resistance to several distinct antimicrobials mediated by the same or one molecular mechanism [71]. Despite their usefulness, biocides have been associated with the induction and co-selection of antimicrobial resistance through co- and cross-resistance mechanisms [72], leading to the use of many of them being discouraged. For example, triclosan and triclocarban had values below the limits of quantification, indicating that these products are now being replaced as recommended by WHO in most household products in South Africa, even though they are not banned in South Africa. However, a previous study had reported higher values in South African water bodies [28], although it has been reported that QACs are more widely used in products within the WWTP catchment area. So the low concentration can be due to the effectiveness of the WWTP and the lack of particulate matter, as most biocides are not entirely solubilised in the aqueous state but can adhere to particulate matter and carbon-rich sediments [73,74]. Nevertheless, given that chemical adhesion to particulate matter was not investigated in the current study, further research is needed to understand the dynamics of these pharmaceuticals in the aquatic environment.

Notably, most of the biocides identified in the current study had higher concentrations than the recommended PNEC value of 0.01 µg/L [75]. The WWTP effluent point has the higher detection concentrations and serves as the point of greatest impact into the WWTP catchment area. This indicates that more effort needs to be applied, especially in the treatment plant, to reduce/remove them from the water.

## 4. Materials and Methods

### 4.1. Study Area and Sampling Site

**Figure 3:**
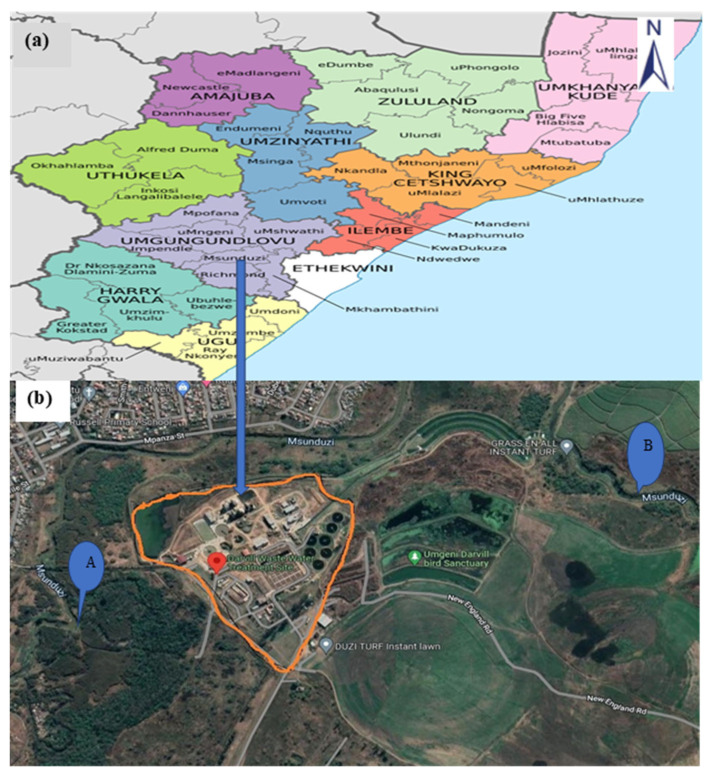
Map of the Wastewater Treatment Plant serving the uMsunduzi Municipality. (**a**) Study area and (**b**) Sampling points for this study (A) Upstream (29°36′02.60″ S 30°25′49.61″ E) (B) Downstream (29°36′27.54″ S 30°27′0.76″ E) (Afzelia Environmental Consultants cc. https://sahris.sahra.org.za/sites/Darvill/WWTP.pdf) (Location of Msunduzi Local Municipality within KwaZulu-Natal Coordinates: 29°37′ S 30°23′ E).

The study was conducted at a WWTP serving the uMsunduzi Municipality that discharges water directly into the uMsunduzi River. The study site and a description of the study area have previously been described [76]. Samples were collected from the final effluent discharged into the river and upstream and downstream from the WWTP discharge point (Figure 3). These points were selected to provide insight into the potential role of the WWTP on the abundance of pollutants in the receiving water body.

### 4.2. Sample Collection, Processing, and Analysis

Samples were collected in February and March 2020. Triplicate grab water samples were collected hourly between 8 am and 2 pm on each sampling day into sterile 500 mL sample bottles. Samples were then pooled into the morning (8–11 am) and evening (1–4 pm) composite samples per sampling round. These samples were immediately frozen and shipped to the Agricultural Research Council, Pretoria (antibiotics and biocides analysis) and WaterLab (Pty) Ltd., Pretoria, South Africa (heavy metals analysis).

### 4.3. Metal Analysis

#### 4.3.1. Digestion of Water Samples for Metal Analysis

The United States Environmental Protection Agency, US EPA method 2007:7 [77] for the digestion of water samples was used with slight modifications. Briefly, 3 mL of concentrated nitric acid (HNO_3_) was added to 50 mL of the water sample in a beaker. The solution was heated at approximately 85 °C to less than 10 mL on a hot plate in a fume hood. The solution was allowed to cool, after which 5 mL of concentrated HNO_3_ was added, and the mixture was heated until digestion was complete. Following cooling, 10 mL of 1:1 HCl: HNO_3_ and 15 mL of deionised water were added. The solution was then heated for 15 min. The walls of the beaker and watch glass were rinsed with deionised water. The solution was filtered using a Whatman No 1 filter paper (Cytiva, MA, USA) into a 100 mL volumetric flask and made up to 100 mL mark with distilled water.

#### 4.3.2. Inductively Coupled Plasma-Optical Emission Spectrometry (ICP-OES) Analysis of Heavy Metals

The digested samples were analysed for heavy metals using inductively coupled plasma-atomic emission spectrometry (ICP-OES) iCAP 6500 DUO (Thermo Scientific, Waltham, MA, USA). The ICP-OES operating conditions used for the determination were 0.7 L/min nebuliser flow, 0.5 L/min auxiliary flow, 1.5 mL/ min sample uptake rate, 1150 W Radio Frequency power, plasma stabilisation time of 10 min and 12 L/min coolant gas flow. Heavy metal determination was achieved using Duo view, while 2-point background correction and three replicates were used to measure the analytical signal. The emission intensities were obtained for the most sensitive lines free of spectral interference. The calibration standards were prepared by diluting the 50 mg/L stock solution of a multi-element standard solution. The detection limit for each metal was obtained following the procedure outlined in the US EPA method 200.7 [77].

#### 4.3.3. Antibiotics and Biocides Analysis

High purity (>97%) analytical standards of targeted analytes were used in this study. All methods were developed and optimised in-house.

#### 4.3.4. Standard Preparation

Water samples were tested for the following sulphonamides: sulfadiazine, sulfathiazole, sulfamethazine, sulphapyridine, sulfamerazine, sulfamethoxypyridazine, sulfachloropyridazine, sulfadoxine, sulfaquinoxaline, sulfamethoxazole and sulfadimethoxine; four tetracyclines including, tetracycline, oxytetracycline, chlortetracycline and doxycycline; two ionophores: lasalocid-A, monesin; one lincosamide: lincomycin; three β-lactams: penicillin, ampicillin and amoxicillin; two anthelmintics: oxfendazole and fenbendazole and one beta agonist: Clenbuterol (Sigma Aldrich, Kempton Park South Africa). For biocides, ammonium acetate and ten biocide standards were used, which included benzyldimethyldecyl-ammonium chloride (BAC-C10), benzyldimethyldodecyl-ammonium chloride (BAC-C12), benzyldimethyltetradecyl-ammonium chloride (BAC-C14), benzyldimethylhexadecyl-ammonium chloride (BAC-C16), benzethonium chloride (BenzEth), didecyldimethyl-ammonium chloride (DDAC), didodecyldimethyl-ammonium bromide (DDAB), dodecyltrimethyl-ammonium bromide (DTAB), triclosan and triclocarban (Sigma-Aldrich, Kempton Park, South Africa).

A 1.0 mg/L multi-residue stock solution containing a mixture of all antibiotic standards was prepared in methanol and kept at −20 °C throughout the analysis. From the stock solution, working standard solutions in the range of 0.5–100 µg L^−1^ were prepared and stored at 4 °C throughout the analysis [78]. A 10-mg/L stock solution containing the combination of the ten biocide standards was prepared in methanol. The solution was kept at −20 °C and was used to prepare 10 µg/L and 1000 ng/L working standard solutions in methanol. These operational standards were further used to prepare an 8-point calibration curve of 1 µg/L to 100 µg/L concentrations.

#### 4.3.5. Sample Preparation and Extraction

Sample preparation and extraction of antibiotics followed a modified solid-phase extraction (SPE) method described previously [78]. Briefly, 500 mL of each water sample was first filtered through a 0.45 µm non-pyrogenic syringe filter (Sarstedt, Numbrecht, Germany) and then centrifuged at 8000 rpm at 4 °C for 10 min. After that, Hydrophile Lipophile Balance (HLB) Solid Phase Extractor (SPE) cartridges (Waters, Milford, MA, USA) were activated and conditioned using 5 mL of methanol, followed by 5 mL deionised water at an approximate flow rate of 5 mL/minute. The samples were passed through the cartridges at a slow flow rate of approximately one drop per second. Next, the cartridges were eluted with 5 mL methanol and 3 mL ethyl acetate. The eluents were then evaporated under a gentle stream of nitrogen to incipient dryness and reconstituted with 1 mL of methanol. These were then filtered into an autosampler vial through a 0.22 µm nylon membrane syringe filter. Three matrix blanks were prepared using deionised water, and a six-point matrix-matched calibration curve, fortified with 0.5 ng/mL, 2 ng/mL, 10 ng/mL, 50 ng/mL, 100 ng/mL, 150 ng/mL, 200 ng/mL and 400 ng/mL were included and processed like the samples.

Five hundred millilitres (500 mL) of water sample were filtered through a 0.45 µm non-pyrogenic syringe filter into a separating funnel to extract biocides. The samples were extracted by solid phase extraction using 6 mL Oasis WCX cartridges (500 mg, 60 µm) (Waters, Milford, MA, USA). First, the cartridge was conditioned with 5 mL each of methanol and deionised water (resistivity of 18.2 Ω). Then, the samples were loaded onto the cartridge at a steady flow rate of approximately 5 mL/min. Next, the cartridge was dried under a vacuum and eluted with 5 mL of 1% formic acid in acetonitrile. The eluate was then concentrated to incipient dryness under a gentle stream of nitrogen at 40 °C and reconstituted in 1 mL of 10 mM ammonium acetate in methanol and filtered through 0.22 µm nylon syringe filter into an HPLC vial.

Deionised water was used to prepare three matrix blanks and an eight-point matrix-matched calibration curve, fortified with 1 ng/mL, 5 ng/mL, 10 ng/mL, 20 ng/mL, 30 ng/mL, 40 ng/mL, 50, ng/mL and 100 ng/mL, following the same procedure as the sample preparation.

#### 4.3.6. Instrumental Analysis

The chromatographic separation of target analytes was achieved using a PerkinElmer^®^ LX-50 UHPLC system (Perkin Elmer, Waltham, MA, USA), equipped with a PerkinElmer^®^ Brownlee SPP (Superficially Porous Particles) C18 (2.7 μm: 100 × 2.1 mm) column. The column oven temperature was set at 40 °C. Separation was attained using 0.1% formic acid in water (solvent A) and acetonitrile (solvent B) as the mobile phases at a constant flow rate of 0.4 mL/min and a sample injection volume of 10 μL. The total run time was 17.2 min, with an equilibration time of 3.5 min between runs.

Chromatographic separation of biocides was achieved using the same PerkinElmer LX-50 UHPLCequipped with a 2.1 × 100mm, 1.7 μm Phenomenex^®^ Kinetex^®^ C_18_ column (Phenomenex, Torrance, CA, USA). The column oven temperature was set at 50 °C, while 10 µL of the sample was injected. The biocides were separated using a linear gradient of 10 mM ammonium acetate in water (solvent A) and methanol (solvent B). These were eluted at a constant flow rate of 0.8 mL/minute. An initial flow of 90% A was held for 5 min, then decreased to 20% for 4.1 min. Following this, the flow was held at 100% B for 3.1 min and then changed to 90% A for 0.1 min, bringing the total run time to 12.3 min. This was followed by 3.5 min of equilibration.

Target antibiotics were identified and quantified using a PerkinElmer^®^ Qsight™ 220 triple quadruple mass spectrometer (TQMS) (PerkinElmer South Africa (Pty) Ltd., Midrand, South Africa) in the positive and negative electrospray ionisation mode. The electrospray voltage was set at 5000 V, with nitrogen as the nebuliser and drying gas at 200 and 120, respectively. The ion source temperature was set at 400 °C, while the Hot-Surface Induced Desolvation (HSID) temperature was set at 320 °C. A time-managed multiple reaction monitoring (MRM) mode was used to acquire the different analytes. At least two MRM transitions were used to positively identify and confirm the presence of the analytes in a sample. The entrance voltages (EV), collision energies (CE) and cell exit potential (CXP) were optimised for each analyte individually. Instrumental data were acquired using Perkin Elmer Simplicity™ 3Q software (version 1.4.1806.29651) (PerkinElmer, Buckinghamshire, UK).

Each biocide was identified and confirmed with a PerkinElmer^®^ Qsight™ 220 TQMS (Waltham, MA, USA) operated in both the positive and negative electrospray ionisation modes. Nitrogen was used as the nebuliser and drying gas for both the positive and negative modes, and these were set at 60 and 120 arbitrary units, respectively. The ion source temperature was kept at 450 °C, and the optimised HSID temperature was set at 320 °C. The electrospray voltage was set at −3000 V for the negative and 2500 V for the positive ionisation modes. A time-managed MRM mode was used to acquire biocides. The CE, EV and CXP were individually optimised in-house for each analyte.

### 4.4. Statistical Analysis

A one-way analysis of variance (ANOVA) with a Games-Howell posthoc test was performed to compare the mean concentrations of the various parameters between the sampling points. Concentrations below the limits of quantification (LOQ) were treated as zero. All statistical analyses were performed using the Statistical Package for the Social Sciences (SPSS) Version 27 and were considered significant at *p* ≤ 0.05.

## 5. Conclusions

This study evaluated the concentration of antibiotics, heavy metals, biocides, and antibiotic-resistant bacteria in a WWTP and its receiving water body and observed that most of the chemical pollutants were higher than the maximum allowable environmental concentrations and the PNEC as applicable. In addition, sulphonamides, β-lactams and tetracyclines were the most detected antibiotics and targeted QACs were detected at significant concentrations, indicating anthropogenic pollution. Furthermore, higher concentrations were observed in the effluent and downstream samples compared to the upstream, suggesting that the WWTP significantly contributed to the downstream pollution. However, while the current study provides valuable information regarding the presence of metals, antibiotics and biocides in the environment, some of which had concentrations above acceptable levels, these results should not be generalised as the sampling regime was not robust enough. For example, some unexplained occurrences like the presence of some pollutants like penicillin in the downstream samples, albeit their absence in the upstream and effluent samples. These could be due to natural occurrences in the environment, although the extremely low concentrations could be due to the sensitivity of the instrumentation used. Nevertheless, more robust studies involving extensive sampling and exposure experiments are needed to ascertain if the observed environmental pollutant concentrations could induce resistance to antimicrobials in previously susceptible bacteria.

## Figures and Tables

**Table 1:** Antibiotic concentrations across sampling sites in the study.

ANTIBIOTICS	PNEC (Resistance Selection) #	Mean Environmental Concentrations (µg/L)		
		Upstream	Effluent	Downstream
Sulfadimidine	NA	0.95	3.92	1.01
Sulfamethazine	NA	0.79	1.74	0.73
Sulfamonomethoxine	NA	0.00	0.00	3.25
Sulfamethoxazole *	16	11.63	144.41	32.33
Sulfapyridine	NA	0.00	5.17	0.32
Oxytetracycline *	0.5	18.79	18.70	18.71
Tetracycline *	1	0.00	10.05	19.73
Doxycycline *	2	5.75	6.68	5.60
Lasalocid A	NA	3.42	1.69	3.34
Monensin	NA	0.80	1.81	1.28
Lincomycin *	2	3.91	7.95	7.81
Penicillin	NA	0.00	0.00	0.55
Amoxycillin *	0.25	62.31	59.73	52.45

Values below the limit of detection are represented as zero. NA represents antibiotics for which PNEC values are unavailable. * Antibiotics with at least one mean value above the PNEC recommended limit. # [1].

## Data Availability

All data have been added to the manuscript and the Supplementary Material.

## Supplementary Materials

The following supporting information can be downloaded at: https://www.mdpi.com/article/10.3390/antibiotics12071166/s1, Table S1: Overall distribution of metals at the study site; Table S2: Comparison of metal concentrations between sampling points; Table S3: Antibiotics distribution across all sampling points; Table S4: Comparison of antibiotic concentrations between sampling points; Table S5: Concentration of biocides at sampling sites.

## References

[1] Bengtsson-Palme J., Larsson D.G.J. (2016). Concentrations of Antibiotics Predicted to Select for Resistant Bacteria: Proposed Limits for Environmental Regulation. Environ. Int..

[2] Martinez J.L. (2009). The Role of Natural Environments in the Evolution of Resistance Traits in Pathogenic Bacteria. Proc. R. Soc. B Biol. Sci..

[3] Larsson D.G.J. (2014). Antibiotics in the Environment. Ups. J. Med. Sci..

[4] Ebele A.J., Oluseyi T., Drage D.S., Harrad S., Abou-Elwafa Abdallah M. (2020). Occurrence, Seasonal Variation and Human Exposure to Pharmaceuticals and Personal Care Products in Surface Water, Groundwater and Drinking Water in Lagos State, Nigeria. Emerg. Contam..

[5] Ergie A.A., Leng Y., Wang J. (2019). Antibiotics and Resistance Genes in Awash River Basin, Ethiopia. Ecohealth.

[6] Kandie F.J., Krauss M., Beckers L.M., Massei R., Fillinger U., Becker J., Liess M., Torto B., Brack W. (2020). Occurrence and Risk Assessment of Organic Micropollutants in Freshwater Systems within the Lake Victoria South Basin, Kenya. Sci. Total Environ..

[7] Madikizela L.M., Ncube S., Chimuka L. (2020). Analysis, Occurrence and Removal of Pharmaceuticals in African Water Resources: A Current Status. J. Environ. Manag..

[8] Matongo S., Birungi G., Moodley B., Ndungu P. (2015). Occurrence of Selected Pharmaceuticals in Water and Sediment of Umgeni River, KwaZulu-Natal, South Africa. Environ. Sci. Pollut. Res..

[9] Segura P.A., Takada H., Correa J.A., El Saadi K., Koike T., Onwona-Agyeman S., Ofosu-Anim J., Sabi E.B., Wasonga O.V., Mghalu J.M. (2015). Global Occurrence of Anti-Infectives in Contaminated Surface Waters: Impact of Income Inequality between Countries. Environ. Int..

[10] Azanu D., Styrishave B., Darko G., Weisser J.J., Abaidoo R.C. (2018). Occurrence and Risk Assessment of Antibiotics in Water and Lettuce in Ghana. Sci. Total Environ..

[11] Moslah B., Hapeshi E., Jrad A., Fatta-Kassinos D., Hedhili A. (2018). Pharmaceuticals and Illicit Drugs in Wastewater Samples in North-Eastern Tunisia. Environ. Sci. Pollut. Res..

[12] Fayiga A.O., Ipinmoroti M.O., Chirenje T. (2018). Environmental Pollution in Africa.

[13] Akele M.L., Kelderman P., Koning C.W., Irvine K. (2016). Trace Metal Distributions in the Sediments of the Little Akaki River, Addis Ababa, Ethiopia. Environ. Monit. Assess..

[14] Diop C., Dewaelé D., Cazier F., Diouf A., Ouddane B. (2015). Assessment of Trace Metals Contamination Level, Bioavailability and Toxicity in Sediments from Dakar Coast and Saint Louis Estuary in Senegal, West Africa. Chemosphere.

[15] Edokpayi J., Odiyo J., Popoola O., Msagati T. (2016). Assessment of Trace Metals Contamination of Surface Water and Sediment: A Case Study of Mvudi River, South Africa. Sustainability.

[16] Agoro M.A., Adeniji A.O., Adefisoye M.A., Okoh O.O. (2020). Heavy Metals in Wastewater and Sewage Sludge from Selected Municipal Treatment Plants in Eastern Cape Province, South Africa. Water.

[17] European Commission (1998). Directive 98/5/EC of the European Parliament and of the Council of 16 February 1998.

[18] De Bruijn J., Hansen B., Johansson S., Luotamo M., Munn S., Musset C., Olsen S., Olsson H., Paya-Perez A., Pedersen F. (2002). Technical Guidance Document on Risk Assessment. Part 1. Part 2.

[19] Chen Z.F., Ying G.G., Lai H.J., Chen F., Su H.C., Liu Y.S., Peng F.Q., Zhao J.L. (2012). Determination of Biocides in Different Environmental Matrices by Use of Ultra-High-Performance Liquid Chromatography-Tandem Mass Spectrometry. Anal. Bioanal. Chem..

[20] Schweizer H.P. (2001). Triclosan: A Widely Used Biocide and Its Link to Antibiotics. FEMS Microbiol. Lett..

[21] Du S., McLaughlin B.A., Pal S., Aizenman E. (2002). In Vitro Neurotoxicity of Methylisothiazolinone, a Commonly Used Industrial and Household Biocide, Proceeds via a Zinc and Extracellular Signal-Regulated Kinase Mitogen-Activated Protein Kinase-Dependent Pathway. J. Neurosci..

[22] Zhao J.L., Zhang Q.Q., Chen F., Wang L., Ying G.G., Liu Y.S., Yang B., Zhou L.J., Liu S., Su H.C. (2013). Evaluation of Triclosan and Triclocarban at River Basin Scale Using Monitoring and Modeling Tools: Implications for Controlling of Urban Domestic Sewage Discharge. Water Res..

[23] Chen Z.F., Ying G.G., Liu Y.S., Zhang Q.Q., Zhao J.L., Liu S.S., Chen J., Peng F.J., Lai H.J., Pan C.G. (2014). Triclosan as a Surrogate for Household Biocides: An Investigation into Biocides in Aquatic Environments of a Highly Urbanised Region. Water Res..

[24] Nuńez O., Moyano E., Galceran M.T. (2004). Determination of Quaternary Ammonium Biocides by Liquid Chromatography-Mass Spectrometry. J. Chromatogr. A.

[25] Montaseri H., Forbes P.B.C. (2016). A Review of Monitoring Methods for Triclosan and Its Occurrence in Aquatic Environments. TrAC-Trends Anal. Chem..

[26] Healy M.G., Fenton O., Cormican M., Peyton D.P., Ordsmith N., Kimber K., Morrison L. (2017). Antimicrobial Compounds (Triclosan and Triclocarban) in Sewage Sludges, and Their Presence in Runoff Following Land Application. Ecotoxicol. Environ. Saf..

[27] Juksu K., Zhao J.L., Liu Y.S., Yao L., Sarin C., Sreesai S., Klomjek P., Jiang Y.X., Ying G.G. (2019). Occurrence, Fate and Risk Assessment of Biocides in Wastewater Treatment Plants and Aquatic Environments in Thailand. Sci. Total Environ..

[28] Lehutso R.F., Daso A.P., Okonkwo J.O. (2017). Occurrence and Environmental Levels of Triclosan and Triclocarban in Selected Wastewater Treatment Plants in Gauteng Province, South Africa. Emerg. Contam..

[29] Li X., Brownawell B.J. (2010). Quaternary Ammonium Compounds in Urban Estuarine Sediment Environments—A Class of Contaminants in Need of Increased Attention?. Environ. Sci. Technol..

[30] Liu W.R., Zhao J.L., Liu Y.S., Chen Z.F., Yang Y.Y., Zhang Q.Q., Ying G.G. (2015). Biocides in the Yangtze River of China: Spatiotemporal Distribution, Mass Load and Risk Assessment. Environ. Pollut..

[31] Mao H., Li H., Li Y., Li L., Yin L., Yang Z. (2020). Four Typical Personal Care Products in a Municipal Wastewater Treatment Plant in China: Occurrence, Removal Efficiency, Mass Loading and Emission. Ecotoxicol. Environ. Saf..

[32] Martínez-Carballo E., González-Barreiro C., Sitka A., Kreuzinger N., Scharf S., Gans O. (2007). Determination of Selected Quaternary Ammonium Compounds by Liquid Chromatography with Mass Spectrometry. Part II. Application to Sediment and Sludge Samples in Austria. Environ. Pollut..

[33] AMR Industry Alliance List of Predicted No-Effect Concentrations (PNECs) (2021). AMR Industry Alliance Antibiotic Discharge Targets.

[34] (2015). SANS South African National Standard (SANS). Drink. Water SANS.

[35] Chetty S., Pillay L. (2019). Assessing the Influence of Human Activities on River Health: A Case for Two South African Rivers with Differing Pollutant Sources. Environ. Monit. Assess..

[36] Awofolu O.R., Mbolekwa Z., Mtshemla V., Fatoki O.S. (2005). Levels of Trace Metals in Water and Sediment from Tyume River and Its Effects on an Irrigated Farmland. Water SA.

[37] Letsoalo M.R., Godeto T.W., Magadzu T., Ambushe A.A. (2018). Quantitative Speciation of Arsenic in Water and Sediment Samples from the Mokolo River in Limpopo Province, South Africa. Anal. Lett..

[38] Perera P.C.T., Sundarabarathy T.V., Sivananthawerl T., Kodithuwakku S.P., Edirisinghe U. (2016). Arsenic and Cadmium Contamination in Water, Sediments and Fish Is a Consequence of Paddy Cultivation: Evidence of River Pollution in Sri Lanka. Achiev. Life Sci..

[39] Xu Y., Tan L., Li Q., Zheng X., Liu W. (2022). Sublethal Concentrations of Heavy Metals Cu^2+^ and Zn^2+^ Can Induce the Emergence of Bacterial Multidrug Resistance. Environ. Technol. Innov..

[40] Stepanauskas R., Glenn T.C., Jagoe C.H., Tuckfield R.C., Lindell A.H., King C.J., McArthur J.V. (2006). Coselection for Microbial Resistance to Metals and Antibiotics in Freshwater Microcosms. Environ. Microbiol..

[41] Li X., Gu A.Z., Zhang Y., Xie B., Li D., Chen J. (2019). Sub-Lethal Concentrations of Heavy Metals Induce Antibiotic Resistance via Mutagenesis. J. Hazard. Mater..

[42] Chen J., Li J., Zhang H., Shi W., Liu Y. (2019). Bacterial Heavy-Metal and Antibiotic Resistance Genes in a Copper Tailing Dam Area in Northern China. Front. Microbiol..

[43] Marshall B.M., Levy S.B. (2011). Food Animals and Antimicrobials: Impacts on Human Health. Clin. Microbiol. Rev..

[44] Michael I., Rizzo L., Mcardell C.S., Manaia C.M., Merlin C., Schwartz T., Dagot C., Fatta-kassinos D. (2012). Urban Wastewater Treatment Plants as Hotspots for the Release of Antibiotics in the Environment: A Review. Water Res..

[45] Batt A.L., Bruce I.B., Aga D.S. (2006). Evaluating the Vulnerability of Surface Waters to Antibiotic Contamination from Varying Wastewater Treatment Plant Discharges. Environ. Pollut..

[46] Mutiyar P.K., Mittal A.K. (2014). Occurrences and Fate of Selected Human Antibiotics in Influents and Effluents of Sewage Treatment Plant and Effluent-Receiving River Yamuna in Delhi (India). Environ. Monit. Assess..

[47] National Center for Biotechnology Information PubChem Compound Summary for CID 5336, Sulfapyridine. https://pubchem.ncbi.nlm.nih.gov/compound/sulfapyridine.

[48] Marks S.L. (2013). Diarrhea. Canine and Feline Gastroenterology.

[49] Ncube S., Nuapia Y.B., Chimuka L., Madikizela L.M., Etale A. (2021). Trace Detection and Quantitation of Antibiotics in a South African Stream Receiving Wastewater Effluents and Municipal Dumpsite Leachates. Front. Environ. Sci..

[50] Fekadu S., Alemayehu E., Dewil R., Van der Bruggen B. (2019). Pharmaceuticals in Freshwater Aquatic Environments: A Comparison of the African and European Challenge. Sci. Total Environ..

[51] K’oreje K.O., Okoth M., Van Langenhove H., Demeestere K. (2020). Occurrence and Treatment of Contaminants of Emerging Concern in the African Aquatic Environment: Literature Review and a Look Ahead. J. Environ. Manage..

[52] Statistics South Africa (Stats SA) (2021). Statistical Release P0302: Mid-Year Population Estimates 2021 (Embargoed until 19th July 2010 10:00).

[53] Agunbiade F.O., Moodley B. (2016). Occurrence and Distribution Pattern of Acidic Pharmaceuticals in Surface Water, Wastewater, and Sediment of the Msunduzi River, Kwazulu-Natal, South Africa. Environ. Toxicol. Chem..

[54] Archer E., Petrie B., Kasprzyk-Hordern B., Wolfaardt G.M. (2017). The Fate of Pharmaceuticals and Personal Care Products (PPCPs), Endocrine Disrupting Contaminants (EDCs), Metabolites and Illicit Drugs in a WWTW and Environmental Waters. Chemosphere.

[55] Ngumba E., Gachanja A., Tuhkanen T. (2016). Occurrence of Selected Antibiotics and Antiretroviral Drugs in Nairobi River Basin, Kenya. Sci. Total Environ..

[56] K’oreje K.O., Vergeynst L., Ombaka D., De Wispelaere P., Okoth M., Van Langenhove H., Demeestere K. (2016). Occurrence Patterns of Pharmaceutical Residues in Wastewater, Surface Water and Groundwater of Nairobi and Kisumu City, Kenya. Chemosphere.

[57] Kairigo P., Ngumba E., Sundberg L.R., Gachanja A., Tuhkanen T. (2020). Occurrence of Antibiotics and Risk of Antibiotic Resistance Evolution in Selected Kenyan Wastewaters, Surface Waters and Sediments. Sci. Total Environ..

[58] Tahrani L., Mehri I., Reyns T., Anthonissen R., Verschaeve L., Khalifa A.B.H., Van Loco J., Abdenaceur H., Mansour H. (2018). Ben UPLC-MS/MS Analysis of Antibiotics in Pharmaceutical Effluent in Tunisia: Ecotoxicological Impact and Multi-Resistant Bacteria Dissemination. Arch. Microbiol..

[59] SCENIHR (Scientific Committee on Emerging and Newly Identified Health Risks) (2009). Assessment of the Antibiotic Resistance Effects of Biocides. Environment.

[60] Javaherdashti R., Akvan F. (2020). Microbiologically Influenced Corrosion (MIC). Failure Modes, Effects and Causes of Microbiologically Influenced Corrosion.

[61] Ceng G.S. (2002). Water and Effluents. Plant Engineer’s Reference Book.

[62] Ünal H. (2018). Antibiofilm Coatings. Handbook of Antimicrobial Coatings.

[63] Kim S., Seo M., Na M., Kim J. (2021). Investigation on Combined Inhalation Exposure Scenarios to Biocidal Mixtures: Biocidal and Household Chemical Products in South Korea. Toxics.

[64] Ferrer I., Furlong E.T. (2001). Identification of Alkyl Dimethylbenzylammonium Surfactants in Water Samples by Solid-Phase Extraction Followed by Ion Trap LC/MS and LC/MS/MS. Environ. Sci. Technol..

[65] Olkowska E., Polkowska Z., Namieśnik J. (2013). A Solid Phase Extraction-Ion Chromatography with Conductivity Detection Procedure for Determining Cationic Surfactants in Surface Water Samples. Talanta.

[66] Berendonk T.U., Manaia C.M., Merlin C., Kassinos D.F., Cytryn E., Walsh F., Bürgmann H., Huovinen P., Stefani S., Schwartz T. (2013). Tackling Antibiotic Resistance: The Environmental Framework. Nat. Rev. Microbiol..

[67] Kreuzinger N., Fuerhacker M., Scharf S., Uhl M., Gans O., Grillitsch B. (2007). Methodological Approach towards the Environmental Significance of Uncharacterized Substances—Quaternary Ammonium Compounds as an Example. Desalination.

[68] Santos M.A.d.O., Vianna M.F., Nishino L.K., Lazarini P.R. (2015). Vestibular Disorders in Bell’s Palsy: A Prospective Study. Rev. Laryngol. Otol. Rhinol..

[69] NRCS Chemical Registration in South Africa, Hazardous Substances Act, GHS. https://csra.freyrsolutions.com/chemical-regulatory-services-in-south-africa.

[70] Wong P.H.P., von Krosigk M., Roscoe D.L., Lau T.T.Y., Yousefi M., Bowie W.R. (2014). Antimicrobial Co-Resistance Patterns of Gram-Negative Bacilli Isolated from Bloodstream Infections: A Longitudinal Epidemiological Study from 2002–2011. BMC Infect. Dis..

[71] Colclough A., Corander J., Sheppard S.K., Bayliss S.C., Vos M. (2019). Patterns of Cross-Resistance and Collateral Sensitivity between Clinical Antibiotics and Natural Antimicrobials. Evol. Appl..

[72] Ruiz L., Alvarez-Ordóñez A. (2017). The Role of the Food Chain in the Spread of Antimicrobial Resistance (AMR).

[73] Tezel U., Pavlostathis S.G. (2015). Quaternary Ammonium Disinfectants: Microbial Adaptation, Degradation and Ecology. Curr. Opin. Biotechnol..

[74] Mc Cay P.H., Ocampo-Sosa A.A., Fleming G.T.A. (2010). Effect of Subinhibitory Concentrations of Benzalkonium Chloride on the Competitiveness of Pseudomonas Aeruginosa Grown in Continuous Culture. Microbiology.

[75] European Chemical Agency (ECHA) (2022). Guidance on the Biocidal Products Regulation: Volume I: Identity of the Active Substance/Physico-Chemical Properties/Analytical Methodology—Parts A+B+C: Information Requirements, Evaluation and Assessment.

[76] Mbanga J., Abia A.L.K., Amoako D.G., Essack S.Y. (2020). Quantitative Microbial Risk Assessment for Waterborne Pathogens in a Wastewater Treatment Plant and Its Receiving Surface Water Body. BMC Microbiol..

[77] EPA (US Environmental Protection Agency) (1994). METHOD 200.7—Determination of Elements and Trace Elements in Water and Wastes by Inductively Coupled Plasma-Atomic Emmission Spectropemtry.

[78] Abafe O.A., Späth J., Fick J., Jansson S., Buckley C., Stark A., Pietruschka B., Martincigh B.S. (2018). LC-MS/MS Determination of Antiretroviral Drugs in Influents and Effluents from Wastewater Treatment Plants in KwaZulu-Natal, South Africa. Chemosphere.

