# Supplementary material for: Antibiotic, Heavy Metal, and Biocide Concentrations in a Wastewater Treatment Plant and Its Receiving Water Body Exceed PNEC Limits: Potential for Antimicrobial Resistance Selective Pressure
Source: Antibiotics (Basel). 2023 Jul 9;12(7):1166. doi: 10.3390/antibiotics12071166 (PMC10376008; doi:10.3390/antibiotics12071166)
Supplement: Supplementary file 1 [file antibiotics-12-01166-s001.zip › antibiotics-2476440-supplementary.pdf]

Supplementary Materials

Table S1: Overall mean metal concentration.

Metal (mg/L)*	AF_Up	AF_Down	AF_Eff	M_Up	M_Down	M_Eff	Mean	SD
Nd	13.068	20.687	67.791	0.088	28.022	36.749	27.734	21.27198
Ca	6.637	8.017	17.358	0.083	16.386	20.375	11.476	7.107189
Mn	4.138	4.671	6.266	0.194	6.956	6.521	4.791	2.288649
La	1.847	2.906	10.051	0.006	3.137	4.413	3.726	3.132304
Sm	4.092	5.328	2.768	0.125	2.18	2.633	2.854	1.613021
Ga	2.198	3.197	0.167	0	0.084	0.097	0.957	1.264778
Al	2.05	2.901	0.269	0	0.1	0.1	0.903	1.141096
Pb	0.072	0.076	2.326	0.086209	0.052	0.155	0.461	0.834439
Na	0.16	0.254	0.122	0	0.025	0.054	0.102	0.086941
Ba	0.074	0.109	0.015	0.002123	0.019	0.026	0.041	0.037834
Ti	0.037	0.05	0.054	0.003637	0.043	0.054	0.04	0.017381
B	0.006	0.006	0.024	0.002582	0.01	0.023	0.012	0.008565
V	0.017	0.017	0.012	0.000593	0.001	0	0.008	0.00754
Co	0.011	0.018	0	0	0	0	0.005	0.007064
Gd	0.01	0.013	0.001	7.48E-05	0.002	0.002	0.005	0.005194
Y	0.01	0.014	0	0.000338	0.001	0	0.004	0.005633
Se	0.002	0.003	0.009	0.000355	0.002	0.004	0.003	0.002787
Mg	0.001	0.001	0.009	0.000265	0.001	0.003	0.002	0.002923
Cu	0.003	0.004	0.007	0	0	0.001	0.002	0.002552
Ni	0.006	0.009	0	0	0	0	0.002	0.003457
Li	0.005	0.008	0	0	0	0	0.002	0.00321
Dy	0.005	0.007	0.001	0	0.001	0	0.002	0.0027
Pd	0.004	0.006	0	0	0	0	0.002	0.00244
Zn	0.004	0.006	0	0	0	0	0.002	0.002287
Os	0.002	0.002	0.002	0.00166	0.001	0	0.001	0.000659
Cr	0.003	0.004	0	0	0	0	0.001	0.001625
Ag	0.002	0	0	0.00172	0.001	0	0.001	0.000695
Si	0.001	0.002	0	0.000436	0.001	0	0.001	0.000572
P	0.002	0.001	0.001	0	0	0	0.001	0.000745
Pr	0.001	0.002	0.001	0	0	0	0.001	0.000639
K	0.001	0.001	0.002	0	0	0.001	0.001	0.000466

Au	0.001	0	0.002	0	0.001	0.001	0.001	0.000539
Rb	0.001	0.002	0	0	0	0	0.001	0.000845
Hf	0.001	0.002	0	0	0	0	0.001	0.000763
Sr	0.001	0.002	0	0	0	0	0.001	0.000745
Hg	0.001	0	0.002	0	0	0.001	0.001	0.000517
Fe	0.001	0.002	0	0	0	0	0	0.000657
**Overall Concentration**								

Key: AF = Afternoon; M = Morning; Up = upstream; Down = Downstream; Eff = Wastewater treatment plant Effluent.

*** As, Be, Cd, Mo, Sb, Se, U were measured but were all below the limit of detection, hence not indicated in the table.**

Table S2: Metal comparison across sampling sites.

	t	df	Sig. (2-tailed)	Mean Difference	95% Confidence Interval of the Difference	
					Lower	Upper
Ag	1.907	2	0.197	0.000804127	-0.00101029	0.0026185
Al	2.373	2	0.141	0.919916667	-0.74804381	2.5878771
Au	2.068	2	0.175	0.000638875	-0.00069051	0.0019683
B	2.019	2	0.181	0.012028704	-0.01360112	0.0376585
Ba	3.397	2	0.077	0.042983961	-0.01146369	0.0974316
Ca	4.971	2	0.038	13.59083333	1.826391044	25.355276
Ce	1.858	2	0.204	0.004808589	-0.00632487	0.0159421
Co	1.943	2	0.191	0.001132621	-0.00137525	0.0036405
Cr	3.152	2	0.088	0.002381375	-0.00086977	0.0056325
Cu	2.211	2	0.158	0.0021793	-0.00206144	0.00642
Dy	1.885	2	0.2	0.000450526	-0.00057779	0.0014788
Fe	2.189	2	0.16	0.97116667	-0.9379916	2.8803249
Ga	2.656	2	0.117	0.004920678	-0.00305012	0.0128915
Gd	1.88	2	0.201	0.000522582	-0.00067364	0.0017188
Hf	1.626	2	0.245	0.000502463	-0.00082701	0.0018319
Hg	2.964	2	0.097	0.000642807	-0.00029022	0.0015758
K	2.461	2	0.133	4.02925	-3.0162263	11.074726
La	1.887	2	0.2	0.002202377	-0.00282064	0.0072254
Li	1.4	2	0.296	0.002441537	-0.00505977	0.0099428
Mg	23.64	2	0.002	5.91725	4.8402823	6.9942177
Mn	3.489	2	0.073	0.1671667	-0.038973	0.373306

Na	2.829	2	0.106	30.92416667	-16.1152003	77.963534
Nd	1.876	2	0.202	0.002365057	-0.00306019	0.0077903
Ni	6.229	2	0.025	0.001457572	0.000450794	0.0024644
Os	5.155	2	0.036	0.00069499	0.0001149	0.0012751
P	1.183	2	0.358	0.461038451	-1.2159026	2.1379795
Pb	1.934	2	0.193	0.001696133	-0.00207821	0.0054705
Pd	3.471	2	0.074	0.000660052	-0.00015821	0.0014783
Pr	1.925	2	0.194	0.000585663	-0.00072326	0.0018946
Rb	2.222	2	0.156	0.003420491	-0.00320225	0.0100432
Se	2.337	2	0.145	0.000788324	-0.00066336	0.00224
Si	10.583	2	0.009	3.22	1.9108357	4.5291643
Sm	1.874	2	0.202	0.000509622	-0.00066059	0.0016798
Sr	6.579	2	0.022	0.043718999	0.015125577	0.0723124
Ti	7.615	2	0.017	0.007875948	0.003425813	0.0123261
V	2.031	2	0.179	0.004272177	-0.00477834	0.0133227
Y	1.922	2	0.194	0.001584638	-0.00196213	0.0051314
Zn	3.138	2	0.088	0.032610162	-0.0121034	0.0773237

Table S3: Antibiotics distribution across all sampling points.

ANTIBIOTICS (µg/L)	Up am 1	Up am 2	Up pm 1	Up pm 2	**Up mean**	EFF am 1	EFF am 2	EFF pm 1	EF pm 2	**Eff Mean**	Down am 1	Down am 2	Down pm 1	Down pm 2	**Down Mean**	Highest Conc
Sulfamethoxazole	18.64	2.8	19.74	5.32	**11.625**	280.26	11.2	286.18	0	**144.41**	54.18	0.62	59.78	14.72	**32.325**	286.18
Amoxycillin	0	120.37	0	128.88	**62.3125**	0	125.94	0	112.99	**59.7325**	0	136.38	0	73.41	**52.4475**	136.38
Tetracycline	0	0	0	0	**0**	40.21	0	0	0	**10.0525**	39.6	0	39.32	0	**19.73**	40.21
Oxytetracycline	37.42	0	37.72	0	**18.785**	36.94	0	37.84	0	**18.695**	37.13	0	37.72	0	**18.7125**	37.84
Lincomycin	0	0	15.65	0	**3.9125**	31.8	0	0	0	**7.95**	8.96	0	22.28	0	**7.81**	31.8
Doxycycline	10.76	0	12.25	0	**5.7525**	13.38	0	13.34	0	**6.68**	10.8	0	11.61	0	**5.6025**	13.38
Sulfapyridine	0	0	0	0	**0**	13.31	0	7.35	0	**5.165**	0	0	1.26	0	**0.315**	13.31
Sulfamonomethoxine	0	0	0	0	**0**	0	0	0	0	**0**	0	0	13	0	**3.25**	13
Albendazole	0	10.88	0	5.8	**4.17**	0	9.37	0	9.6	**4.7425**	0	8.85	0	7.8	**4.1625**	10.88
Sulfadimidine	0	0	3.79	0	**0.9475**	9.78	0	5.89	0	**3.9175**	0	0	4.03	0	**1.0075**	9.78
Lasalocid A	6.84	0	6.84	0	**3.42**	0	0	6.74	0	**1.685**	6.66	0	6.69	0	**3.3375**	6.84
Monensin	0	0	3.21	0	**0.8025**	3.74	0	3.5	0	**1.81**	1.75	0	3.37	0	**1.28**	3.74
Sulfamethazine	0	0	3.16	0	**0.79**	3.63	0	3.31	0	**1.735**	0	0	2.93	0	**0.7325**	3.63
Penicillin	0	0	0	0	**0**	0	0	0	0	**0**	0	0	2.2	0	**0.55**	2.2

Key: pm = Afternoon; am = Morning; Up = upstream; Down = Downstream; Eff = Wastewater treatment plant Effluent. 1 and 2 represent the first and second samples at the respective sampling times

Table S4: Comparison of antibiotic concentrations between sampling points.

	t	df	Sig. (2-tailed)	Mean Difference	95% Confidence Interval of the Difference	
					Lower	Upper
Sulfamethoxazole	2.67	21	0.014	54.80136	12.12353	97.4792
Amoxycillin	6.894	21	0	80.22621	56.02486	104.4276
Tetracycline	2.45	21	0.023	8.145227	1.231493	15.05896
Oxytetracycline	3.715	21	0.001	13.63962	6.004322	21.27492
Lincomycin	2.568	21	0.018	5.618409	1.068349	10.16847
Doxycycline	3.684	21	0.001	4.433788	1.930761	6.936815
Sulfapyridine	2.022	21	0.056	1.767424	-0.05064	3.585493
Sulfamonomethoxine	1.572	21	0.131	1.280303	-0.41392	2.974527
Albendazole	6.85	21	0	6.041515	4.207439	7.875591
Sulfadimidine	2.56	21	0.018	1.690227	0.317114	3.06334
Lasalocid A	3.285	21	0.004	2.101742	0.771174	3.432311
Monensin	3.169	21	0.005	0.995682	0.342272	1.649092
Sulfamethazine	2.852	21	0.01	0.855985	0.231842	1.480128
Penicillin	1.572	21	0.131	0.216667	-0.07005	0.503382

Table S5. Concentration of biocides at sampling sites.

BIOCIDE (µg/L)	Up am 1	Up am 2	Up pm 1	Up pm 2	Up Mean	EFF am 1	EFF am 2	EFF pm 1	EFF pm 2	Eff Mean	Down am 1	Down am 2	Down pm 1	Down pm 2	Down Mean	Highest conc
BAC 10	0.97	0	0.76	0.95	**0.67**	0.72	0	0.61	1.05	**0.595**	0	1.08	1.14	0	**0.555**	1.08
BAC 12	0	1.64	0	29.58	**7.805**	0	0	0	9.01	**2.2525**	0	0	1.24	6.19	**1.8575**	29.58
BAC 14	0	0	0	0	**0**	0	0	0	0	**0**	0	0	2.42	0	**0.605**	2.42
BAC 16	0	0	1.37	0	**0.3425**	0	0	0.5	0.5	**0.25**	0	0	0.39	0	**0.0975**	1.37
BenthEZ	0.06	0	0.09	0.11	**0.065**	0.07	0.07	0	0.12	**0.065**	0	0.04	0.04	0.06	**0.035**	0.12
DDAC	0	0	0	0	**0**	0	0	0.77	0	**0.1925**	0	0.83	0	0.83	**0.415**	0.83
DADMAC 10	0	14.53	0	0	**3.6325**	0	14.65	4.53	0	**4.795**	0	0	0	10.26	**2.565**	14.65
DADMAC 12	0	0	0	0.22	**0.055**	0	0	0	0	**0**	0	1.2	0.01	0.02	**0.3075**	1.2
